# A combined morphological and genetic survey of helminths in the European green toad *Bufotes viridis* (Laurenti, 1768) from eastern Slovakia

**DOI:** 10.1017/S0031182025100966

**Published:** 2025-11

**Authors:** Kristián Gulyás, Romana Gašparovičová, Monika Balogová, Natália Pipová, Petr Papežík, Jessica Hriňáková, Dalibor Uhrovič, Marcel Uhrin, Michal Benovics

**Affiliations:** 1 Department of Zoology, Faculty of Science, Pavol Jozef Šafárik University in Košice, Košice, Slovakia; 2 Department of Animal Physiology, Faculty of Science, Pavol Jozef Šafárik University in Košice, Košice, Slovakia; 3 Department of Zoology, Faculty of Natural Sciences, Comenius University in Bratislava, Bratislava, Slovakia; 4 Department of Botany and Zoology, Faculty of Science, Masaryk University, Brno, Czech Republic; 5 Unit for Environmental Sciences and Management, North-West University, Potchefstroom, South Africa

**Keywords:** amphibia, helminth diversity, host-parasite dynamics, integrative taxonomy, nematoda, toads

## Abstract

Accurate characterization of helminth communities in amphibian hosts is essential for understanding host-parasite dynamics in changing environments. This study presents an integrative parasitological survey of *Bufotes viridis* populations in eastern Slovakia, using both morphological and molecular methods. A total of 61 road-killed individuals collected across 13 localities were examined for helminth presence. Only nematodes were detected, encompassing 3 families, Rhabdiasidae, Molineidae and Cosmocercidae. Four nematode species were recorded, *Rhabdias rubrovenosa, Oswaldocruzia filiformis, O. ukrainae* and *Aplectana linstowi*, and a further unidentified *Cosmocerca* species. Notably, *R. rubrovenosa* seems to be a new or previously misidentified helminth species found in Slovakia. Cosmocercidae represented the most abundant family, while Molineidae occurred scarcely. Each species was characterized genetically – for the members of Rhabdiasidae and Cosmocercidae, partial 18S rDNA, complete ITS1, complete 5.8S rRNA, complete ITS2 and partial 28S rDNA sequences were amplified, whereas for representatives of Molineidae, partial COI sequences were obtained. These results underscore the utility of combining molecular and morphological tools in helminth biodiversity studies and provide updated baseline data on nematode infections in *B. viridis* within an anthropogenically influenced landscape. Despite visual patterns indicating differences in the community compositions of nematode families between urban and rural localities, multivariate analyses testing revealed no significant differences.

## Graphical abstract

**Figure:**
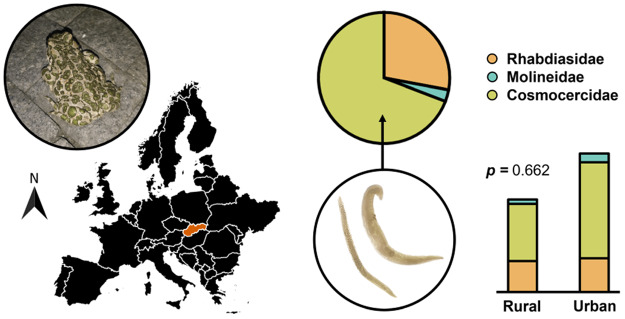

## Introduction

In amphibian research, increasing attention is being directed toward understanding the dynamics of host-parasite interactions (Johnson and Buller, 2011). Despite the ongoing decline of amphibian populations worldwide, there is only limited knowledge about their parasites. Investigating the patterns and key factors that shape the composition of parasite communities provides valuable insights into the ecology of both amphibians and parasites (Herczeg et al. 2021).

Amphibians, like other vertebrate groups, are affected by both microparasites (e.g. viruses, bacteria, fungi), protists (e.g. flagellata, amoebae) and macroparasites (e.g. helminths, arthropods and leeches), which are among the most characteristic and commonly studied organisms affecting amphibian hosts (Densmore and Green, 2007; Herczeg et al. 2021). They frequently serve as intermediate or definitive hosts for numerous parasitic taxa, including members of the phyla Platyhelminthes Claus, 1887, Acanthocephala Koelreuter, 1771, and Nematoda Rudolphi, 1808 (Bower et al. 2019).

Parasitic infections are influenced by the interaction between host-specific characteristics and ecological processes. Traits such as body size, age, immune function, physiology, diet and behaviour shape both exposure and susceptibility to parasites (Stromberg, 1997; Thieltges et al. 2008; Araujo et al. 2015; Rakus et al. 2017). Larger or longer-lived hosts, for instance, may provide more space and resources for colonization and are generally exposed to infective stages for longer durations (Poulin, 1997). In addition to host characteristics, ecological attributes, such as host density, dispersal ability and interspecific interactions, also enhance the potential for contact with infective stages and facilitate parasite transmission (Brian and Aldridge, 2022). Species with extensive geographic distributions also tend to encounter a greater diversity of parasites and may enable cross-species transmission via habitat overlap (Poulin, 2004). Parasite assemblages within hosts are not fixed but reflect ongoing colonization and extinction processes, mirroring patterns observed in island biogeography theory (Poulin, 2004). In recent years, attention has also turned toward the evolutionary history of host species as a key factor shaping parasite diversity (Morand, 2015). The evolutionary duration of exposure to parasitic organisms can vary between different host species, influencing not only their potential for parasite acquisition and transmission but also the degree of host-parasite co-adaptation (Campião et al. 2015). Over time, this may lead to the development of strong host specificity, especially in cases where parasites have evolved alongside a particular host species. Such long-term associations may be reflected in the presence of parasite taxa that are highly specialized or even exclusive to certain hosts (Poulin and Keeney, 2008).

The European green toad (*Bufotes viridis* (Laurenti, 1768)) is a medium sized toad and suitable model for parasitological research due to its ecological plasticity and wide geographic distribution across Europe and western Asia (Speybroeck et al. 2016; Dufresnes et al. 2019). Recent phylogenetic studies have shown that *B. viridis*, which was once regarded as a single species, is a potential complex comprising several distinct, though closely-related, species across its range (Dufresnes et al. 2019). This species inhabits a variety of environments, ranging from natural steppes and wetlands to highly urbanized areas (Stöck et al. 2008; Speybroeck et al. 2016; Vargová et al. 2023). As a result, it is exposed to a broad spectrum of endo- and ectoparasitic taxa. So far, studies on endohelminths of the *B. viridis* complex in Europe have been conducted in several countries, including Czechia (Vojtková and Vojtek, 1975; Vojtková, 1976, 1979, 1980, 1989), Belarus (Shimalov and Shimalov, 2001), Ukraine (Marushchak et al. 2024), Moldova (Gherasim and Erhan, 2024) and European Russia (Kirillova et al. 2023). In Asia, research has been carried out in Turkey (Yildirimhan, 1999; Düşen and Oğuz, 2010; Düşen et al. 2010), Jordan (Al-Sorakhy and Amr, 2003), Iraq (Mohammad et al. 2010), Iran (Rakhshandehroo et al. 2017) and Uzbekistan (Vashetko and Siddikov, 1999). To date, helminthological investigations have recorded 51 species infecting the *B. viridis* complex, belonging to 28 families, encompassing 3 phyla, Platyhelminthes, Acanthocephala and Nematoda (Supplementary Table 1). In *B. viridis sensu stricto* (defined by Dufresnes et al. 2019), which occurs in central and eastern Europe and throughout Russia, 40 helminth species had been recorded so far.

In this study, we examined the helminthofauna of *B. viridis* in eastern Slovakian populations, using morphological and molecular approaches and assessed whether the composition of parasite communities in this species differs between urban and rural localities. Previous studies dealing with the helminthofauna of *B. viridis* in the Slovak Republic were mainly conducted in the second half of the last century (Prokopič, 1957; Kozák, 1966, 1969a, 1969b; Prokopič and Křivanec, 1975; Vojtková, 1976) and relied solely on the morphological characteristics of the species. A more recent contribution to this topic was published by Gulyás et al. (2025) but focused solely on the genus *Oswaldocruzia* from different host species including *B. viridis* and provided the first molecular evidence of *O. ukrainae* in Slovakia. Because modern molecular methodologies are gradually revealing the limitations of traditional morphological identification, and because of the large time gap since the earliest research conducted in the previous century, we expected that the helminth communities of *B. viridis* identified in this study would differ from the those revealed in previous records. Additionally, we hypothesized that *B. viridis* populations inhabiting urban and rural environments would exhibit differences in parasite community composition, reflecting contrasting ecological conditions.

## Material and methods

### Material collection

**Figure 1.**
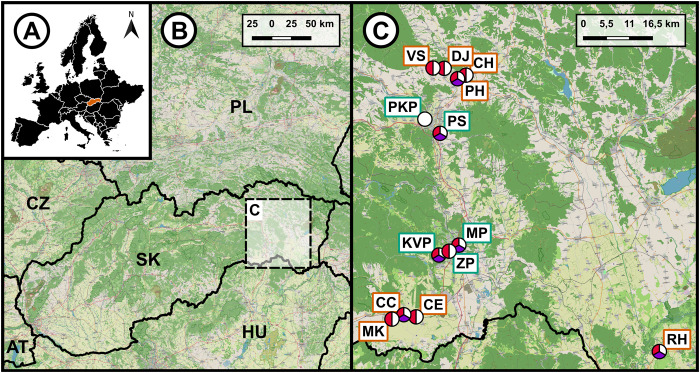
Map of the sampling localities in eastern Slovakia. (A) The position of Slovakia in Europe; (B) Slovakia with the highlighted examined area; (C) the examined localities with the respective abbreviations of the localities (the names corresponding to the abbreviations are showed in Table 2), the colours in the circles indicate the parasite families recorded at each locality (white: rhabdiasidae; purple: molineidae; red: cosmocercidae). A brown border around the site abbreviation represents rural localities, and a green border represents urban localities. SK, Slovakia; CZ, Czechia; PL, Poland; HU, Hungary; AT, Austria; UA, Ukraine.

A total of 61 individuals of *B. viridis* (frozen cadavers) from 13 localities across eastern Slovakia were analysed for parasite presence (Figure 1, Supplementary Table 2). The localities were subjectively classified according to the level of anthropogenic development. Localities with less built-up areas situated in villages were classified as rural, while densely built-up areas situated within cities were classified as urban (Figure 1C). Prior to examination, the frozen toads were thawed. Following dissection of the toads, the internal organs (lungs, heart, liver, kidneys, spleen, small and large intestines) were placed in a saline solution and examined under a stereomicroscope. The only helminths found in *B. viridis* were nematodes. All the nematodes were removed from the above-mentioned organs and preserved in 70% or 96% ethanol for subsequent morphological and molecular analyses. The identification to families was carried out on the basis of the localization of the individuals (Rhabdiasidae in the lungs, Molineidae in the small intestine and Cosmocercidae in the large intestine), and on the basic of the morphological characteristics of each family (Vojtková, 1976; Anderson et al. 2009). The identification of samples to species belonging to the families Rhabdiasidae and Cosmocercidae was carried out by a combination of molecular and morphological approaches (for morphological identification, see, e.g. Vojtková, 1976; Baker, 1980; Kuzmin, 2013; Ikromov et al. 2023; Velázquez-Brito et al. 2023). Microscopic examination was performed, and morphological measurements and photomicrographs taken using a Leica DM 2500 microscope equipped with an Axiocam 208 colour digital camera. All measurements are presented in millimetres, unless stated otherwise. The basic quantitative parameters of parasite populations, including prevalence, mean abundance, and the minimum and maximum infection intensities, were estimated for each parasite family according to the methodology described by Bush et al. (1997). Epidemiological parameters were determined at the family level due to inadequacies in the quality of the material and the inability to reliably distinguish between all species in each family. Prevalence was defined as the percentage of toads infected by a particular parasite family, while mean abundance referred to the average number of parasite individuals of a given family per host, including both infected and uninfected individuals. To support interpretation of the quantitative data, 95% confidence intervals were calculated for mean abundance following the recommendations of Rózsa et al. (2000). Given the relatively small host sample sizes per population, the bias-corrected and accelerated bootstrap (BCa) method was employed using QPweb software (Reiczigel et al. 2019) to calculate the confidence intervals for mean abundance values. The identification of samples from the Molineidae family was based solely on molecular analyses given the low occurrence of these nematodes in the examined host samples.

### Genomic DNA extraction, amplification, sequencing

From the individuals of Cosmocercidae and Rhabdiasidae, hologenophores were created by cutting out pieces of less morphological importance. In case of Rhiabdiasidae, small portions of the posterior ends of individuals were cut off and saved in 96% ethanol for molecular analyses. In the case of the Cosmocercidae family, individuals were cut into thirds, and the middle portion was saved for molecular analyses. The remaining portions of the nematodes were mounted on slides and covered in a mixture of glycerine and water (in the ratio 3:7) and cleared by gradually increasing the volume of glycerol (according to Moravec, 2013). Genomic DNA from specimens (or their parts) stored in 96% ethanol was extracted using the DNeasy Blood & Tissue Kit (Qiagen, Hilden, Germany) following the respective manufacturer’s protocol. After DNA extraction, polymerase chain reactions (PCR) were carried out. Either PCR was performed in a total volume of 15 μl containing 1 U of DreamTaq DNA polymerase (Thermo Fisher Scientific, Waltham, MA, USA), 1 × Taq Buffer, 1.5 mM MgCl2, 300 µM of each dNTP, 0.5 μM of each primer, 2 μl of DNA template (corresponding to 20 ng/μl) and nuclease-free water (for Molineidae), or it was carried out in a total volume of 20 μl containing 4 μl of FIREPol Master Mix Ready to Load (Solis BioDyne OÜ, Tartu, Estonia), 0.5 μM of each primer, 2 μl of DNA template and nuclease-free water (for Rhabdiasidae and Cosmocercidae). The amplified gene segments for each nematode family, the primers used for the amplification, the PCR conditions and the protocols which were followed during the PCR are listed in Table 1. PCR products were detected by electrophoresis in 1.5% agarose gels stained with GoodView (SBS Genetech, Beijing, China). Amplified products were purified using EPPiC Fast (A&A Biotechnology, Gdansk, Poland), following the manufacturer’s protocol. Sequencing was performed in both directions using the PCR primers. Commercial services provided by Macrogen Europe (Amsterdam, Netherlands) were used for sequencing.

**Table 1.** Amplified gene regions, primers, PCR conditions and protocols used for each nematode family

Family	Targeted genomic region(s)	PCR conditions	Primers used	Reference/protocol
Rhabdiasidae	Partial 18S rDNA, complete ITS1, 5.8S rDNA & ITS2, partial 28S rDNA	95 °C 3 min; 40 × (95 °C 30 s, 53 °C 30 s, 72 °C 2 min); 72 °C 10 min	Rift (forward, 5′-GCGGCTTAATTTGACTCAACACGG-3′)	Tkach et al. (2014)
			1500 R (reverse, 5′-GCTATCCTGAGGGAAACTTCG-3′)	
Molineidae	Partial COI	95 °C 3 min; 40 × (95 °C 30 s, 53 °C 30 s, 72 °C 1 min); 72 °C 7 min	JB3 (forward, 5’-TTTTTTGGGCATCCTGAGGTTTAT-3’)	Bowles et al. (1992)
			JB4.5 (reverse, 5’-TAAAGAAAGAACATAATGAAAATG-3’)	
Cosmocercidae	Partial 18S rDNA, complete ITS1, 5.8S rDNA & ITS2, partial 28S rDNA	95 °C 15 min; 35 × (95 °C 30 s, 55 °C 30 s, 72 °C 1 min); 72 °C 10 min	18S_F (forward, 5’-TTGATTACGTCCCTGCCCTTT-3’)	Vrain et al. (1992)
			26S_R (reverse, 5’-TTTCACTCGCCGTTACTAAGG-3’)	

### Sequence dataset assembly and phylogenetic analyses

**Figure 2.**
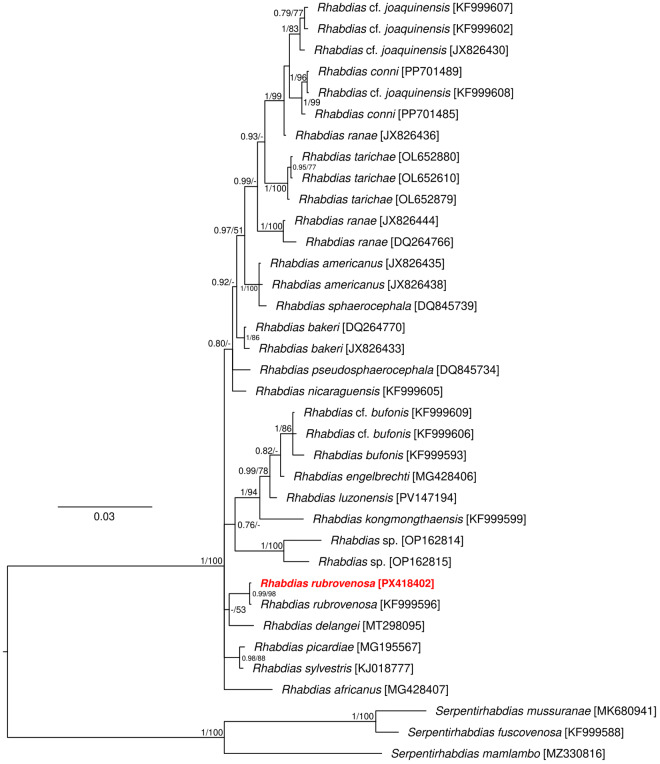
Phylogenetic tree of 33 sequences of *Rhabdias* spp. reconstructed by Bayesian inference. The tree is based on a 1551 bp-long alignment of the targeted genomic region (partial 18S rDNA, complete ITS1, complete 5.8S rRNA, complete ITS2 and partial 28S rDNA) and rooted using *Serpentirhabdias* orthologous sequences as the outgroup. Values at the nodes indicate posterior probabilities (>70) from the Bayesian inference, and bootstrap values (>50) from the maximum likelihood analysis. Lower values are shown as dashes (–). The length of branches represents the number of substitutions per site. The newly-generated sequence of specimen collected from *B. viridis* is in red. GenBank accession numbers are in brackets at the taxa name.

In order to assess the phylogenetic position of the obtained nematode species, additional orthologous sequences from congeners or different phylogenetically close species were obtained from GenBank (accession numbers are included within phylogenetic trees, Figures 2, 4, 5). The sequences were aligned by means of the fast Fourier transform algorithm implemented in MAFFT (Katoh, 2002), using the G-INS-i refinement method. For the *Rhabdias* spp. and Cosmocercidae spp. dataset, the general time-reversible (GTR; Lanave et al. 1984) model was applied for the entire length of the alignment, including both a gamma distribution and the proportion of invariable sites. For the *Oswaldocruzia* spp. dataset built of COI sequences, the data were treated as codon partitioned, and a GTR model was selected independently for each position within the codon, including both a gamma distribution and the proportion of invariable sites. Phylogenetic trees were constructed using Bayesian inference (BI) and Maximum likelihood (ML) approaches in MrBayes 3.2 (Ronquist et al. 2012) and RAxML 8.1.12 (Stamatakis 2006, 2014), respectively. BI analysis used the Metropolis-coupled Markov chain Monte Carlo algorithm with 2 parallel runs of 1 cold and 3 hot chains, and was run for 10^6^ generations, sampling trees every 100 generations. The initial 30% of all saved trees were discarded as ‘burn-in’ after checking that the standard deviation split frequency fell below 0.01. The convergence of the runs and the parameters of individual runs were checked using Tracer v. 1.7.1 (Rambaut et al. 2018). Posterior probabilities for each tree node were calculated as the frequency of samples recovering a given clade. The clade bootstrap support for ML trees was assessed by simulating 10^3^ pseudoreplicates.

### Statistical analysis

To assess potential differences in parasite community composition between urban and rural localities, a non-metric multidimensional scaling (NMDS) analysis based on Bray–Curtis dissimilarities was performed. Prior to ordination, parasite abundance data were Hellinger-transformed using the decostand() function from the *vegan* package. The NMDS was conducted in 2 dimensions (k = 2), with a maximum of 100 iterations to ensure convergence. The final solution yielded a low stress value (0.017), indicating a good fit of the ordination.

To test for significant differences in parasite community structure between urban and rural localities, permutational multivariate analysis of variance (PERMANOVA) based on Bray–Curtis dissimilarities was used. The test was implemented with 1000 permutations using the adonis2() function in *vegan*.

All analyses and visualizations were conducted in R, version 4.4.1 (R Core Team, 2024) using the packages *vegan* (Oksanen et al. 2025), *ggplot2* (Wickham, 2016), *ggrepel* (Slowikowski, 2024), *ggalt* (Rudis et al. 2025) and *ggpubr* (Kassambara, 2025).

## Results

### Diversity and distribution of helminths in Bufotes viridis

All 61 examined toads were infected with nematodes belonging to at least 1 out of 5 families. A total of 1834 nematodes were extracted from the toads, out of which 512 were of the family Rhabdiasidae, 53 of Molineidae and 1269 of Cosmocercidae (Supplementary Table 2). The presence of nematodes belonging to all 3 families was recorded in only 6 localities (CC, KVP, MP, PH, PS and RH) (Figure 1, Table 2). The Molineidae were the least represented family within the examined toads, as they were not recorded in 7 out of the 13 examined localities (CE, DJ, CH, ZP, MK, PKP and VS). Cosmocercidae exhibited the highest abundance among all collected nematodes, and were not only recorded from toads in PKP. The only family which was represented in all 13 localities was Rhabdiasidae, with a prevalence ranging from 20% (1/5) to 100% (5/5). Of the localities where more than 1 host individual was examined, the highest mean abundance was recorded in the Cosmocercidae family in KVP, with an abundance of 42.10 and a mean intensity of infection of 84.20 (3–205). Molecular and morphological approaches unequivocally confirmed the presence of 5 nematode species parasitizing *B. viridis* in the studied area.

**Table 2.** List of collection localities with geographic coordinates, sample sizes of *B. Viridis* individuals examined and epidemiological parameters of nematode families recorded in each population

Locality	Loc_ID	Loc_Type	Coordinates	NO_Host	Families	P	A	CL	I
Cestice	CE	Rural	48.590383	5	Rhabdiasidae	100.0%	14.0	4.6–23.4	14.0 (3–31)
			21.100551		Molineidae	0.0%	–	–	–
					Cosmocercidae	80.0%	4.8	1.2–8.8	6.0 (2–12)
Čečejovce	CC	Rural	48.598886	5	Rhabdiasidae	80.0%	16.4	2.2–52.8	20.5 (1–66)
			21.062267		Molineidae	20.0%	0.2	0–0.4	1.0 (1)
					Cosmocercidae	100.0%	13.4	6.2–20.6	13.4 (3–25)
Demjata	DJ	Rural	49.107776	1	Rhabdiasidae	100.0%	3.0	–	3.0 (3)
			21.311242		Molineidae	0.0%	–	–	–
					Cosmocercidae	100.0%	118.0	–	118.0 (118)
Chmeľovec	CH	Rural	49.082935	1	Rhabdiasidae	100.0%	2.0	–	2.0 (2)
			21.371885		Molineidae	0.0%	–	–	–
					Cosmocercidae	100.0%	2.0	–	2.0 (2)
Košice – KVP	KVP	Urban	48.715139	10	Rhabdiasidae	30.0%	5.8	1.0–15.2	19.3 (10–33)
			21.211511		Molineidae	40.0%	0.9	0.2–2.1	2.3 (1–4)
					Cosmocercidae	50.0%	42.1	14.2–99.9	84.2 (3–205)
Košice – City Park	MP	Urban	48.723888	6	Rhabdiasidae	100.0%	23.5	9.8–49.3	23.5 (5–68)
			21.265415		Molineidae	66.7%	6.0	0.5–20.2	9.0 (1–30)
					Cosmocercidae	100.0%	27.2	17.0–39.0	27.2 (10–47)
Košice – Zuzka’s Park	ZP	Urban	48.718835	5	Rhabdiasidae	100.0%	10.4	7.0–13.6	10.4 (6–15)
			21.238002		Molineidae	0.0%	–	–	–
					Cosmocercidae	100.0%	38.8	19.4–67.6	38.8 (9–86)
Mokrance	MK	Urban	48.593335	5	Rhabdiasidae	100.0%	13.4	6.2–20.2	13.4 (5–24)
			21.022273		Molineidae	0.0%	–	–	–
					Cosmocercidae	60.0%	10.4	1.2–26.6	17.3 (3–35)
Podhorany	PH	Rural	49.083212	4	Rhabdiasidae	50.0%	2.0	0–4.5	4.0 (2–6)
			21.356049		Molineidae	25.0%	1.0	0–2	4.0 (4)
					Cosmocercidae	100.0%	31.8	4.5–79.5	31.8 (3–100)
Prešov – City square	PKP	Urban	49.006469	5	Rhabdiasidae	40.0%	1.4	0–3.4	3.5 (2–5)
			21.224027		Molineidae	0.0%	–	–	–
					Cosmocercidae	0.0%	–	–	–
Prešov – Šváby	PS	Urban	48.971488	5	Rhabdiasidae	40.0%	2.0	0–4	5.0 (4–6)
			21.266167		Molineidae	20.0%	0.2	0–0.4	1.0 (1)
					Cosmocercidae	60.0%	4.8	0.6–9.4	8.0 (3–13)
Rad site Hrušov	RH	Rural	48.435857	5	Rhabdiasidae	20.0%	1.0	–	1.0 (1)
			21.861214		Molineidae	20.0%	2.0	–	2.0 (2)
					Cosmocercidae	20.0%	1.0	–	1.0 (1)
Vyšný Slivník	VS	Rural	49.112080	4	Rhabdiasidae	25.0%	2.8	0–5.5	11.0 (11)
			21.275219		Molineidae	0.0%	–	–	–
					Cosmocercidae	75.0%	19.0	4.8–33.8	25.3 (18–39)

Locality = the names of the examined localities; Loc_ID = abbreviation of the examined localities; Loc_Type = the defined type of locality (rural/urban); NO_Host = number of processed *B. viridis* individuals in each locality; P = prevalence; A = mean abundance; CL = 95% confidence limits for the population mean abundance; I = mean intensity of infection with range in parentheses.

### Taxonomic and morphological-genetic description

Order: Rhabditida Chitwood, 1933

Family: Rhabdiasidae Railliet, 1916

Genus: *Rhabdias* Stiles & Hassall, 1905

Species: *Rhabdias rubrovenosa* (Schneider, 1866) Semenov, 1929

The final alignment was built of 38 sequences (also including 3 *Serpentirhabdias* Tkach et al. 2014 orthologous sequences as an outgroup for rooting the phylogenetic tree and 32 *Rhabdias* sequences retrieved from GenBank) and spanned 1551 unambiguously aligned nucleotide positions. Both phylogenetic analyses (BI and ML) generated trees with congruent topologies, and therefore only the BI tree is presented with posterior probabilities and bootstrap support values (Figure 2). The phylogenetic tree confirmed the presence of *R. rubrovenosa* in the studied green toads, having 100% identity with the conspecific sequence in GenBank (KF999596) obtained from the specimen from Ukraine (host *B. viridis*). *Rhabdias rubrovenosa* is phylogenetically closely related to *R. delangei* Kuzmin, Svitin, Harnoster & du Preez, 2020, which is a species described from South African anurans, and the 2 species also share a similar morphological feature (i.e. a non-functional rectum and anus). The phylogenetic relationship of these 2 species to congeners was not resolved.

**Figure 3.**
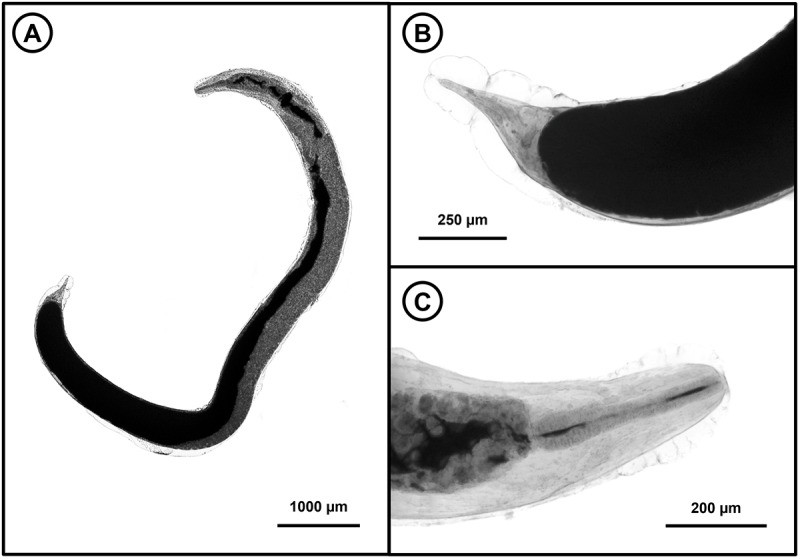
Photomicrographs of *Rhabdias rubrovenosa*. (A) Entire hermaphroditic parasitic adult; (B) caudal region of the specimen showing absent anus; (C) anterior end of the specimen with visible oesophagus.

Subsequent morphometric analysis further confirmed the identity of all collected *Rhabdias* specimens as *R. rubrovenosa* (Figure 3). On the basis of measurements of 20 individuals, the body length ranges from 4.891 to 11.828. The vulva is situated 2.869 to 8.301 from the anterior end (all obtained measurements are provided in Table 3). The tail length ranges from 0.207 to 0.500, and the anus is absent. All the above-mentioned parameters correspond with previous morphometric records of the species (Kuzmin, 2013) and clearly distinguish it from *R. bufonis* (Schrank, 1788) Stiles & Hassall, 1905 (Table 3). The presence of this nematode was confirmed at all the examined localities.

**Table 3.** Morphometric comparison of *Rhabdias rubrovenosa* and *R. bufonis* (mm), the only 2 *Rhabdias* species documented in *Bufotes viridis* to date

	*R. rubrovenosa*	*R. rubrovenosa*	*R. bufonis*	*R. bufonis*	*R. bufonis*	*R. bufonis*
	Parasitic ♀	Parasitic ♀	Parasitic ♀	Parasitic ♀	Parasitic ♀	Parasitic ♀
BoL	4.891–11.828	4.000–11.900	3.600–13.800	4.800–7.520	3.880–13.000	6.100–7.490
OeL	0.270–0.441	0.307–0.423	0.377–0.682	0.352–0.400	0.324–0.589	0.391–0.444
NDA	0.132–0.239	0.141–0.216	–	0.160–0.176	0.133–0.241	0.180–0.210
VDA	2.869–8.301	2.050–5.600	1.950–8.110	2.240–3.520	2.200–7.000	3.120–3.980
Anus	Absent	Absent	Present	Present	Present	Present
TaL	0.207–0.500	0.208–0.498	0.212–0.549	0.280–0.320	0.192–0.498	0.242–0.353
Ref	(present study)	(Kuzmin, 2000)	(Vojtková, 1976)	(Wang et al. 1992)	(Kuzmin, 2013)	(Zeng et al. 2024)

BoL = total body length; OeL = oesophagus length; NDA = neural ring distance from the anterior end; VDA = vulva distance from the anterior end; TaL = tail length; Ref = reference to the respective publication.

Family: Molineidae Skryabin & Schulz, 1937

Genus: *Oswaldocruzia* Travassos, 1917

Species: *Oswaldocruzia filiformis* (Goeze, 1782) Skrjabin & Schultz, 1952

Species: *Oswaldocruzia ukrainae* Iwanitzky, 1928

**Figure 4.**
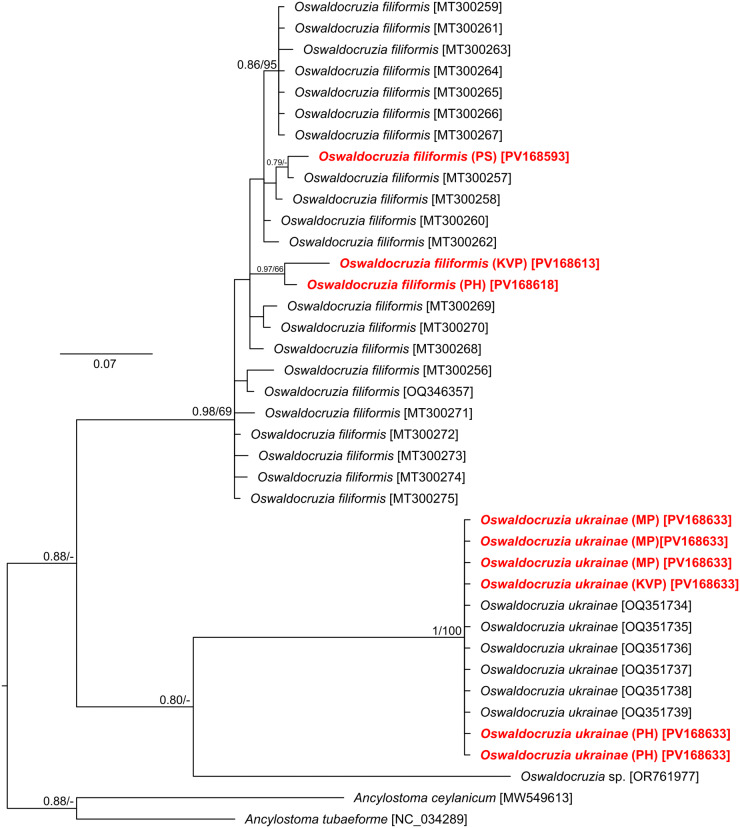
Phylogenetic tree of 37 COI sequences of 3 *Oswaldocruzia* species reconstructed by Bayesian inference. The tree is based on a 370 bp-long alignment and rooted using *Ancylostoma tubaeforme* and *A. ceylanicum* as the outgroups. Each *Oswaldocruzia filiformis* represents a unique haplotype. Values at the nodes indicate posterior probabilities (>70) from the Bayesian inference, and bootstrap values (>50) from the maximum likelihood analysis. Lower values are shown as dashes (–). The length of branches represents the number of substitutions per site. Sequences of specimens collected from *B. viridis* are in red. GenBank accession numbers are in brackets at the taxa name.

The final alignment was built of 39 sequences (also including 2 *Ancylostoma* orthologous sequences as an outgroup for rooting the phylogenetic tree and 27 *Oswaldocruzia* sequences retrieved from GenBank) and spanned 370 unambiguously aligned nucleotide positions. Both phylogenetic analyses (BI and ML) generated trees with congruent topologies, and therefore only the BI tree is presented with posterior probabilities and bootstrap support values (Figure 4). The phylogenetic analysis confirmed the presence of 2 *Oswaldocruzia* species in the examined *B. viridis* toads. The first was *O. filiformis*, where 3 different haplotypes were recorded. The second one was *O. ukrainae*, among which all analysed specimens were genetically identical and also identical with the sequences retrieved from GenBank obtained from specimens from Russia (hosts *B. viridis*). *O. filiformis* was confirmed at 3 localities (KVP, PH and PS), while *O. ukrainae* was detected at 5 localities (CC, KVP, MP, PH and RH).

Family: Cosmocercidae Railliet, 1916

**Figure 5.**
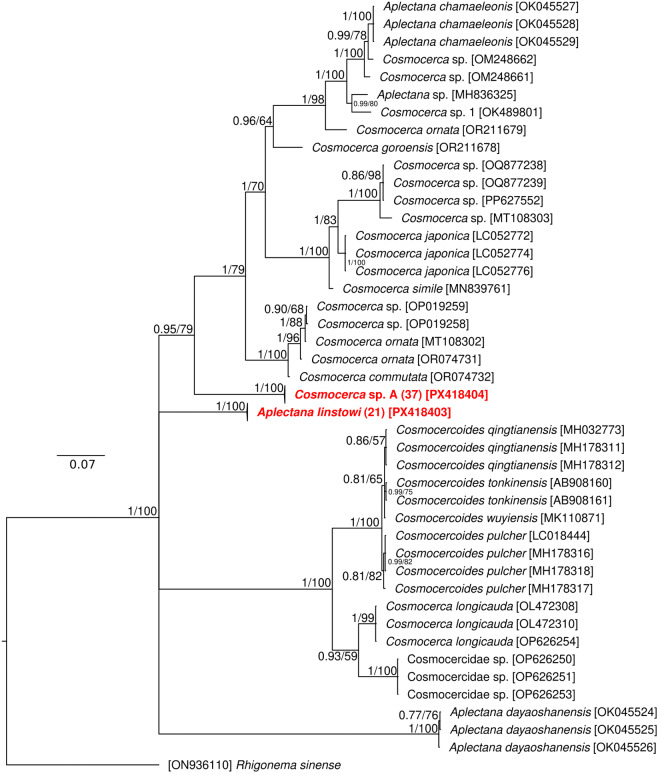
Phylogenetic tree of 99 sequences of Cosmocercidae species reconstructed by Bayesian inference. The tree is based on a 1149 bp-long alignment of the targeted genomic region (partial 18S rDNA, complete ITS1, complete 5.8S rRNA, complete ITS2 and partial 28S rDNA) and rooted using *Rhigonema sinense* as the outgroup. Values in parenthesis represent the number of identical sequences in the collapsed branch. Values at the nodes indicate posterior probabilities (>70) from the Bayesian inference, and bootstrap values (>50) from the maximum likelihood analysis. Lower values are shown as dashes (–). The length of branches represents the number of substitutions per site. Newly generated sequences of specimens collected from *B. viridis* are in red. GenBank accession numbers are in brackets at the taxa name.

The final alignment was built of 100 sequences (also including the *Rhigonema sinense* orthologous sequence as an outgroup for rooting the phylogenetic tree and 41 sequences of various representatives of Cosmocercidae retrieved from GenBank) and spanned 1149 unambiguously aligned nucleotide positions. Both phylogenetic analyses (BI and ML) generated trees with congruent topologies, and therefore only the BI tree is presented with posterior probabilities and bootstrap support values (Figure 5). The phylogenetic analysis confirmed the presence of 2 Cosmocercidae species from different genera in examined *B. viridis* toads. No intraspecific genetic variability was recorded among conspecific specimens; therefore, the branches including all conspecific sequences are collapsed in the phylogenetic tree. The phylogenetic reconstruction also indicated paraphyletic groupings within the family, and sequence comparisons with existing GenBank data failed to reliably resolve the identification of the specimens at the species level. Consequent species identification based on morphological characteristics confirmed the presence of *Aplectana linstowi* and an unidentified species of *Cosmocerca* (hereinafter referred to as *Cosmocerca* sp. A).

Genus: *Aplectana* Railliet & Henry, 1916

Species: *Aplectana linstowi* Yorke & Maplestone, 1926

**Figure 6.**
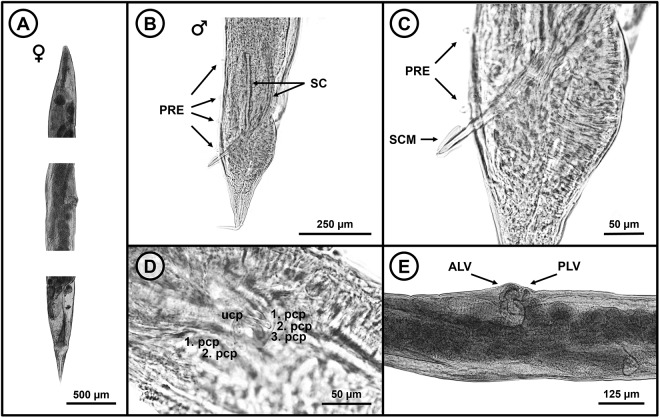
Photomicrographs of *Aplectana linstowi*. (A) Anterior, middle and posterior part of a female; (B) caudal region of a male individual; (C) close-up picture of the male spicules showing the apical depression and thin membrane; (D) anterior lip of anus of a male specimen with 1 unpaired and 3 pairs of caudal papillae; (E) female vulva with extremely swollen anterior lip. PRE, preanal caudal papillae; SC, spicules; SCM, spicule membrane; PCP, paired caudal papillae; UCP, unpaired caudal papillae; ALV, anterior lip of vulva; PLV, posterior lip of vulva.

From the obtained measurements within this study (Table 4), the male specimens (based on measurements of 50 individuals) exhibit a body length ranging from 2.186 to 3.860. The nerve ring is situated 0.123 to 0.343 from the anterior extremity. On the ventral side 4 to 6 pairs of large caudal preanal papillae are present (Figure 6B). The length of the spicules measures between 0.104 and 0.263, while the gubernaculum ranges from 0.040 to 0.121. The distal end of the spicules has an apical depression covered by a thin membrane (Figure 6C). On the anterior lip of the anus, 3 pairs and 1 unpaired caudal papillae are present (Figure 6D). The females (based on 80 specimens) display a body length of between 2.752 and 5.497. The vulva is positioned in the posterior half of the body at a distance of 1.675–3.298 from the anterior extremity. The anterior lip of the vulva is extremely swollen (Figure 6E). All the morphological features are in line with previous observations (Table 4) confirming the species identity as *A. linstowi*. The markedly swollen anterior lip of the vulva and the apical depression on the spicules clearly distinguish it from other species within the genus *Aplectana* (Baker, 1980). The presence of this species was confirmed at 11 localities: CE, CC, DJ, KVP, MP, MK, PH, PS, RH, VS and ZP.

**Table 4.** Comparative morphometric parameters of *Aplectana linstowi* (mm) from the current and previously published studies

	*A. linstowi*		*A. linstowi* (*A. kutassi*)		*A. linstowi*	
	♂	♀	♂	♀	♂	♀
BoL	2.186–3.860	2.752–5.497	2.200–3.700	5.390–6.330	2.500–2.900	3.600–4.500
BoW	0.109–0.257	0.143–0.477	0.210	0.350–0.440	–	–
OeL	0.376–0.522	0.401–0.692	0.428–0.486	0.580–0.590	0.476–0.524	0.527–0.541
BuL	0.064–0.112	0.066–0.125	0.084–0.091	0.112–0.119	–	–
BuL	0.044–0.111	0.031–0.141	0.084–0.091	–	–	–
NDA	0.123–0.343	0.113–0.228	0.220–0.290	0.230–0.260	0.220–0.250	0.247–0.265
VuC	–	Extremely swollen anterior lip	–	–	–	Extremely swollen anterior lip
VPo	–	Posterior half	–	Posterior half	–	–
VDA	–	1.675–3.298	–	3.490–3.870	–	2.200–2.800
EgS	–	Oval	–	–	–	Oval
NPA	4–6	–	5	–	7–9	–
SpC	Apical depression + membrane	–	Apical membrane	–	Apical depression + membrane	–
SpL	0.104–0.263	–	0.220–0.240	–	0.175–0.204	–
GuL	0.040–0.121	–	0.063–0.070	–	0.053–0.055	–
Ref	(present study)		(Vojtková, 1976)		(Baker, 1980)	

BoL = total body length; BoW = body width; OeL = oesophagus length; BuL = bulbus length; BuW = bulbus width; NDA = neural ring distance from the anterior end; VuC = vulva characteristics; VPo = vulva position; VDA = vulva distance from the anterior end; EgS = egg shape; NPA = number of preanal papillae; SpC = spicule characteristics; SpL = spicule length; GuL = gubernaculum length; Ref = reference to the respective publication.

Genus: *Cosmocerca* Diesing, 1861

Species: *Cosmocerca* sp. A

**Figure 7.**
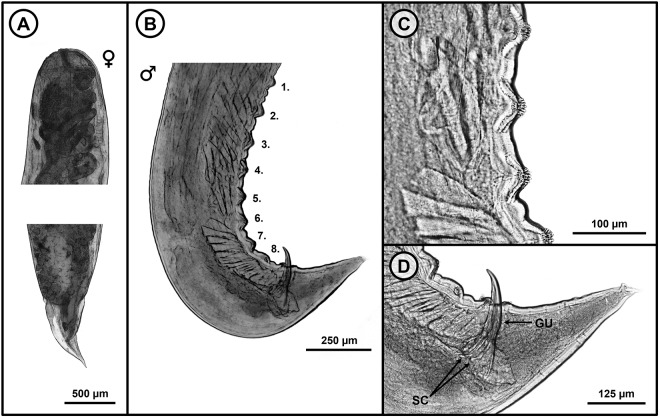
Microphotographs of *Cosmocerca* sp. A. (A) Anterior and posterior part of females; (B) caudal region of male individuals with 8 pairs of plectanes; (C) detailed picture of the plectanes; (D) close-up of the gubernaculum and spicules. GU, gubernaculum; SC, spicules.

From the obtained measurements within this study (Table 5), the male individuals (based on measurements of 5 specimens) exhibit a body length ranging from 3.654 to 5.196. On the ventral side of the caudal region of the males, 7–8 plectanes can be observed (Figure 7B, C). The spicules measures between 0.166 and 0.209. The gubernaculum is prominent, measuring 0.230–0.265 (Figure 7D). The female’s body (based on 26 specimens) exhibits a length of 4.118–8.312. The vulva is positioned in the anterior half of the body at a distance of 1.235–3.547 from the anterior extremity. Even though all the morphological analyses correspond with the morphology of *C. commutata* (Diesing, 1851) Diesing, 1861 from previous studies (Table 5), the molecular analyses did not reveal a resemblance with previously obtained sequences of this species, leaving this species unidentified. This species was detected at 9 localities: CC, DJ, CH, MP, MK, PH, PS, VS and ZP.

**Table 5.** Comparative morphometric parameters of *Cosmocerca* sp. A, with all the *Cosmocerca* species from the Palearctic region (mm)

	*Cosmocerca* sp. A		*C. commutata*		*C. commutata*		*C. commutata*		*C. ornata*		*C. ornata*		*C. ornata*		*C. ornata*	*C. longicauda*	*C. longicauda*	*C. sardiniae*	*C. banyulensis*
	♂	♀	♂	♀	♂	♀	♂	♀	♂	♀	♂	♀	♂	♀	♂	♂	♂	♂	♂
BoL	3.654–5.196	4.118–8.312	3.100–4.500	5.400–6.700	4.300–6.100	4.500–6.900	4.030–4.390	4.760–6.350	1.500–3.010	5.050–10.270	1.600–2.200	2.750–5.110	1.100–1.800	2.900–3.800	1.100–2.800	2.720–3.810	2.900	3.410–4.180	0.970
BoW	0.374–0.562	0.282–0.956	0.350–0.460	0.510–0.720	0.340–0.530	0.390–0.800	0.408–0.448	0.449–0.816	0.095–0.258	0.286–0.653	0.110–0.180	0.210–0.330	0.100–0.160	0.240–0.360	–	0.163–0.326	–	–	–
OeL	0.503–0.563	0.451–0.792	0.470–0.560	0.480–0.610	0.420–0.580	0.490–0.640	0.558–0.571	0.680–0.762	0.276–0.449	0.435–0.666	0.290–0.350	0.470–0.520	0.240–0.310	0.400–0.550	–	0.408–0.554	–	–	–
BuL	0.101–0.137	0.066–0.214	0.110–0.140	0.120–0.160	0.114–0.136	0.100–0.160	0.105–0.114	0.156	0.048–0.066	0.120–0.150	0.060–0.070	0.070–0.080	0.047–0.061	0.053–0.091	–	0.076–0.105	–	–	–
BuW	0.084–0.134	0.073–0.199	0.086–0.103	0.095–0.107	–	–	0.120	0.156–0.210	0.051–0.060	0.105–0.156	0.052–0.059	0.090–0.100	0.041–0.051	0.098–0.109	–	0.084–0.105	–	–	–
NDA	0.138–0.271	0.107–0.199	0.175–0.181	0.194–0.223	–	–	0.210–0.225	0.250–0.272	0.144–0.225	0.201–0.313	0.150–0.190	0.120–0.170	0.118–0.174	0.147–0.182	–	0.225–0.279	–	–	–
VPo	–	Anterior half	–	–	–	–	–	Mid to anterior half	–	Mid region	–	Mid region	–		–	–	–	–	–
VDA	–	1.235–3.547	–	4.340–4.740	–	–	–	–	–	1.290–5.580	–	1.390–2.620	–	1.460–1.960	–	–	–	–	–
EgS	–	Oval	–	–	–	–	–	Oval	–	Oval	–	Oval	–		–	–	–	–	–
SpL	0.166–0.209	–	0.210–0.230	–	–	–	0.180	–	Rudimentary	–	0.072–0.077	–	0.101–0.106	–	Rudimentary	–	0.092	0.375	0.010
GuL	0.230–0.265	–	0.170–0.220	–	0.200–0.250	–	0.186–0.213	–	0.102–0.132	–	0.082–0.086	–	0.091–0.101	–	0.375	0.219–0.258	0.190	0.260–0.303	0.080
NPl	7–8	–	8	–	–	–	8	–	5	–	5	–	5	–	5	5–7	6	4	5–6
LAl	Present	Present	Present	Present	–	–	Present	Present	Present	Present	Present	Present	Present	Present	Present	Present	Present	Absent	Present
Ref	(Present study)		(Ikromov et al. 2023)		(Yildirimhan, 1999)		(Vojtková, 1976)		(Vojtková, 1976)		(Sou et al., 2018)		(Ikromov et al. 2023)		(Skrjabin et al., 1961)	(Vojtková, 1976)	(Skrjabin et al., 1961)	(Ricci, 1987)	(Chabaud and Campana, 1955)

BoL = total body length; BoW = body width; OeL = oesophagus length; BuL = bulbus length; BuW = bulbus width; NDA = neural ring distance from the anterior end; VuC = vulva characteristics; VPo = vulva position; VDA = vulva distance from the anterior end; EgS = egg shape; NPA = number of preanal papillae; SpC = spicule characteristics; SpL = spicule length; GuL = gubernaculum length; Ref = reference to the respective publication.

### Parasite community structure across urban and rural localities

**Figure 8.**
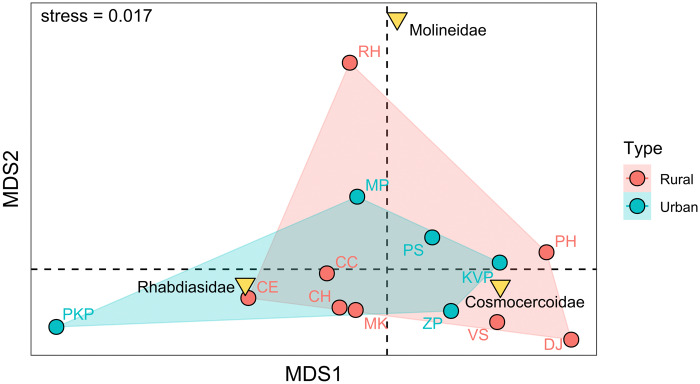
Non-metric multidimensional scaling (NMDS) ordination of parasite communities based on Hellinger-transformed abundance data and Bray–Curtis dissimilarity. Each point represents a sampling locality, colour-coded by locality type (red: rural; blue: urban). Parasite taxa (triangles) are plotted as species scores, with positions indicating their contribution to differences among localities.

NMDS broadly clustered the localities according to their urban or rural classification, with substantial overlap (Figure 8). Overlaying the centroids of the nematode families revealed slight habitat-related associations. Molineidae were primarily associated with rural localities along the second axis, while centroids of the other 2 families lacked any clear pattern. Despite some evident visual patterns in the case of the Molineidae family, statistical testing using PERMANOVA did not reveal a significant difference in parasite community composition between urban and rural localities (Table 6). Only 3.3% of the variation in community structure was explained by the urban–rural classification, indicating high within-group variability and the low explanatory power of locality type alone.

**Table 6.** Results of PERMANOVA (adonis2) testing the effect of locality type (urban vs. rural) on parasite community composition based on Bray–Curtis dissimilarities and Hellinger-transformed abundance data. Significance was assessed using 1000 permutations

Source	Df	Sum of Squares	R^2^	F	*P*-value
Loc_Character	1	0.02199	0.03288	0.374	0.662
Residual	11	0.64675	0.96712		
Total	12	0.66874	1.00000		

## Discussion

### Overview of helminth diversity in Bufotes viridis

This study presents the first combined morphological and molecular assessment of the helminth fauna of *B. viridis* in Slovakia. Notably, only nematode parasites were detected, in contrast to earlier studies from the region (i.e. eastern Slovakia – Prokopič 1957; Kozák 1969a, 1969b), which consistently reported a broader spectrum of endohelminths, including platyhelminths (monogeneans, trematodes) and acanthocephalans, although only with low prevalence and infection intensities. This absence may be the result of the more complex life cycles of trematodes and acanthocephalans, which require 1 or more intermediate hosts before infecting their definitive amphibian host (Kennedy 2006; Koprivnikar et al. 2012). It is possible that suitable intermediate hosts were absent or significantly scarce at the examined localities (as was recorded in the proximal region by Oros and Hanzelová (2009), thereby preventing these endohelminths from completing their life cycles. In addition, long-term environmental changes such as changes in water management practices, urban expansion and pollution over the past 5 decades may have altered the abundance and distribution of intermediate host populations in the examined region (Calegaro-Marques et al. 2014), further reducing the transmission success of parasites with indirect life cycles. In Slovakia, such changes have been well documented. Izakovičová et al. (2022) showed that from the 1970s onward, rapid urban expansion, industrial growth, and transport infrastructure fragmented natural ecosystems and reduced ecological stability, particularly in lowland regions. Similarly, Pazúr and Bolliger (2017) reported that between 1990 and 2012, Slovakia underwent one of the fastest rates of land conversion in central Europe, with large areas of agricultural and semi-natural land transformed into urban and industrial zones. In contrast, nematodes, which have mostly direct life cycles and are less dependent on intermediate hosts, are likely to be less affected by such environmental changes (Sures et al. 2025). Moreover, nematodes exhibit a range of adaptations that enhance their resilience in extreme or fluctuating conditions, including a protective cuticle, a chemically inert exoskeleton and the ability to enter developmental dormancy during adverse periods (McSorley 2003), which could also have contributed to the exclusive detection of nematodes in the present study. It is also possible that flatworms were either overlooked or completely damaged, as only collected cadavers were examined in this study.

While earlier surveys have documented up to 12 nematode species parasitizing *B. viridis* in the examined region (Prokopič, 1957; Kozák, 1969a, 1969b), only 5 were confirmed in the present study. This difference in observed nematode diversity can be connected to a combination of ecological change and methodological variation. Although numerous nematode species possess direct life cycles, their distribution is still under the influence of environmental variables such as habitat disturbance, host density and microclimate (Bradley and Altizer, 2006; Brian and Aldridge, 2022). Urbanization and habitat alteration likely reduce habitat suitability for a number of species, to the advantage of generalist or more tolerant taxa. Coba-Males et al. (2023), who studied the use of road-killed animals as a source of genetic material for biodiversity research, stated that DNA quality and quantity decline with time since death, particularly in amphibians, while environmental exposure further compromises sample viability. Given this, the methodological differences between this study and previous ones, such as the use of road-killed specimens, may have contributed to the observed differences, as post-mortem degradation and restricted ecological representation could have led to the loss or underrepresentation of fragile or host-specific parasites, thereby biasing the results toward more resilient and abundant nematode taxa. Earlier studies were based solely on morphological identification, which could have led to an overestimation of parasite richness, while the combined morphological and molecular approach in this study likely improved the accuracy of identification but at the cost of overlooking rare or degraded specimens (Ondrejicka et al. 2014). For example, *O. iwanitzkyi* Sudarikov (1951) was first described by Iwanitzky (1940) and later revised by Sudarikov (1951). The only other reports of this species are those of Kozák (1969a, 1973). Since then, it has not been recorded again. Theoretically, all these records could refer to *O. ukrainae*, as *B. viridis* was the only host species in which it was found. This possibility was also suggested by Kirillova et al. (2023). A similar case is presented by Vodiasova et al. (2022), who demonstrated that combining morphological and molecular methods in identifying *Ligophorus* Euzet & Suriano, 1977 species parasitizing fish improved taxonomic accuracy but revealed lower species richness, highlighting how morphology alone can overestimate diversity because of cryptic variation and limited diagnostic resolution. The herein provided parasite report is thus likely the result of both improved taxonomic resolution and ecological filtering over time.

## Nematode population composition of *Bufotes viridis* in Slovakia

The most widespread species identified in this study, and the only species representing the Rhabdiasidae family, was *Rhabdias rubrovenosa*. Notably, this species has not been previously recorded in Slovakia, where the only *Rhabdias* species reported from *B. viridis* until now was *R. bufonis* (in the eastern part by Prokopič (1957) and Kozák (1969a), and in the west by Vojtková (1976). The presence of *R. rubrovenosa* was confirmed at all examined localities, with a prevalence ranging from 20% to 100% and a mean abundance of between 1 and 23.5 individuals per host (Table 2). These values fall within the broad epidemiological variability reported for *Rhabdias* spp. across different host species and regions. For example, in Turkey, *R. bufonis* has been reported with a prevalence of 51% and an intensity of infection of 1–53 in *B. sitibundus* (Pallas, 1771) (previously *B. viridis*), and with a prevalence of 17% and an intensity of infection of 1 in *Bufo bufo* (Linnaeus, 1758) (Düşen, 2011). In *Amietophrynus regularis* (Reuss, 1833), the prevalence of *R. bufonis* ranged from 35% with an intensity of infection of 3–5 (Morsy et al. 2018) to 37% with an intensity of infection 0–6 (Ibrahim, 2008). In Slovak amphibians, *R. bufonis* showed a prevalence of 19% and an intensity of infection of up to 148 specimens per host frog (Kozák, 1969a). Both phylogenetic (Figure 2) and morphological analyses (Table 3) conducted in this study clearly confirmed the presence of *R. rubrovenosa*, while *R. bufonis* was entirely absent among the examined toads. Although the possibility of historical misidentification cannot be ruled out, it seems unlikely that multiple previous authors would have consistently misclassified the species. *R. rubrovenosa* was first described by Schneider (1866) as *Leptodera rubrovenosa*. Semenov (1929) later redescribed the species from *B. bufo* (provided under the synonym *Bufo cinereus* Schneider, 1799) and reassigned it to the genus *Rhabdias*. The first description in *B. viridis* was by Mazurmovich (1951) in the vicinity of Kyiv and Kaniv. Later, it was reported by Kuzmin (2000) as a common parasite of *B. viridis* in Ukraine and southwestern Russia. The absence of prior records from Slovakia is likely due to the host specificity of *R. rubrovenosa*, which appears to be primarily associated with *B. viridis*. Given its distinct morphological features, such as the absence of an anus, *R. rubrovenosa* should be easily distinguished from *R. bufonis* even without molecular tools. However, the 2 species are morphologically quite similar, and in young specimens the reduction of the rectum and anus in *R. rubrovenosa* is less pronounced, making misidentification possible. The current findings therefore most likely represent cases that were previously overlooked or misidentified as *R. bufonis*, while this study provides the first confirmed record of *R. rubrovenosa* in Slovakia.

In contrast, members of the Molineidae family were relatively rare and restricted to only a few localities. Their presence was confirmed at 5 localities, with a prevalence ranging from 20% to 67% and a mean abundance of between 0.2 and 6 individuals per host (Table 2). In previous studies, *O. filiformis*, a well-documented representative species of the family, was reported with a prevalence as low as less than 4% in 236 *Pelophylax ridibundus* (Pallas, 1771) specimens from Lake Hazar in Turkey (Saglam and Arıkan, 2006). Similarly, Mannela et al. (2015) reported a 9% prevalence and a mean intensity of 0.82 in 130 *Hoplobatrachus tigerinus* (Daudin, 1802) from India. In contrast, a systematic study by Kirillova et al. (2020) in Russia revealed higher prevalence levels (48–83%) and mean abundances of 2 to 6 worms per host among 260 different anuran and reptilian hosts across the studied region. These findings highlight the substantial variability in infection rates within the Molineidae family, suggesting that the values recorded in the present study are well within the expected range. In the current study, molecular data confirmed the presence of 2 *Oswaldocruzia* species, *O. filiformis* and *O. ukrainae*, whose co-occurrence at certain localities (KVP & PH) underscore the cryptic diversity that may be overlooked in morphology-only studies. These identifications were based on mitochondrial COI sequences and confirmed by comparison with reference sequences from previously described species (Kirillova et al. 2020, 2023). Morphological or morphometric analyses were not conducted due to the low abundance of the recovered individuals and the condition of the relatively low number of collected males, which are used for species identification in this genus (see Kirillova et al. 2020). *O. filiformis* is a widely-distributed nematode known to parasitize a range of amphibian and reptilian hosts across Europe and parts of Asia (Griffin, 1988; Kirillova and Kirillov, 2020). In contrast, *O. ukrainae* seems to be more restricted in its host range, this including *Bombina bombina* (Linnaeus, 1758), *B. variegata* (Linnaeus, 1758), *B. bufo, B. viridis* and *Rana arvalis* Nilsson, 1842 (Vojtková, 1976), while most of its records are linked with *B. viridis* (Vojtková et al. 1972; Baker, 1981; Kirillova et al. 2020, 2023), indicating possible host-specificity to this amphibian host.

Among the 3 nematode families identified in this study, representatives of the family Cosmocercidae were the most abundant, with more than 1200 specimens sampled and identified at 11 out of the 13 examined localities. The molecular analyses revealed the presence of 2 genetically-distinct taxa within the examined specimens. However, species-level identification using only molecular data was impossible, as none of the obtained sequences matched existing records in GenBank. Despite this, morphological analysis allowed for the reliable identification of *A. linstowi*. Previous historical records (Kozák, 1969a) have noted the occurrence of this species in *B. viridis* in eastern Slovakia, under the synonym *Aplectana kutassi (*Iwanitzky, 1940). Of the 2 identified taxa, *A. linstowi* was more prevalent, occurring at 11 localities, while the unidentified *Cosmocerca* sp. A was found at 9. Members of the Cosmocercidae family have been described to utilize direct life cycles, typically transmitted via faecal–oral routes (*Aplectana*) or through skin or eye penetration by infective larvae (*Cosmocerca*) (Anderson, 2000; Kirillova and Kirillov, 2021). These transmission modes are particularly effective in moist environments commonly used by amphibians (Anderson, 2000). *Cosmocerca* sp. A morphologically closely resembled *C. commutata*, particularly in the number of plectane pairs (7–8), a key diagnostic feature for *Cosmocerca* species within the Palearctic region (Bursey et al. 2015; Velázquez-Brito et al. 2023). Additional morphometric information, such as total body length, spicule and gubernaculum length, and the presence of lateral alae further supported the alignment with *C. commutata* (Table 5). However, the absence of sequence conformity with published *C. commutata* references prevented definitive identification, and thus the specimens are kept as *Cosmocerca* sp. A in the current study. The uncertainty in taxonomic classification underlines the necessity to enhance molecular reference databases for the Cosmocercidae family, which is noted for its high cryptic diversity, morphological variation and apparent polyphyly within the genera (Ikromov et al. 2023).

### Urban–rural comparison of the nematode assemblages

One of the goals of this study was to assess whether urban and rural habitats differ in parasite community composition. While visual interpretation of the NMDS plot (Figure 8) showed only a weak pattern indicating the association of the family Molineidae with rural localities, the observed pattern was not supported statistically. PERMANOVA analysis (Table 6) revealed no significant differences in community composition between urban and rural localities (*P* = 0.662), suggesting that habitat type alone accounts for only a small portion of the observed variation. These findings contrast with those of Jacinto-Maldonado et al. (2022), who reported a clear reduction in parasite species richness in urban environments, suggesting that habitat alteration and urban expansion may negatively impact parasite diversity. As highlighted by Brian and Aldridge (2022), parasite communities are shaped by multiple, simultaneously acting ecological and biological factors. Even though the absence of statistically significant differences between urban and rural localities may reflect high within-group variability and potentially the influence of additional unmeasured factors, such as microhabitat quality, host density or local climatic conditions, it is more likely to be the result of limited sample size, the condition of the processed host animals, low parasite diversity and coarse locality classification. This may explain why a simple and subjective urban–rural classification and limited sample size is insufficient to fully account for the observed patterns in the helminth distribution of *B. viridis* among the examined localities.

## Conclusion

This study presents a (1) complete revision of the helminth fauna of *B. viridis* in its distribution range and (2) new records and molecular and morphological data on *B. viridis* parasites from eastern Slovakia. It also highlights the importance of combining morphological and molecular approaches in parasite biodiversity assessments. Although no clear differences in the patterns of parasite assemblages were observed between urban and rural localities, our results provide support for continued investigations into the ecological and evolutionary processes underlying helminth assemblage patterns in anthropogenically modified habitats.

## Supporting information

Gulyás et al. supplementary material Gulyás et al. supplementary material

Gulyás et al. supplementary material Gulyás et al. supplementary material

## Data Availability

The data supporting the conclusions of this study are included in the article. All new DNA sequences of parasites obtained during this study were deposited in GenBank and are available under accession numbers PX418402-PX418404.

## Supplementary material

The supplementary material for this article can be found at https://doi.org/10.1017/S0031182025100966.
