# A combined morphological and genetic survey of helminths in the European green toad *Bufotes viridis* (Laurenti, 1768) from eastern Slovakia – ADDENDUM

**DOI:** 10.1017/S0031182025101170

**Published:** 2026-06

**Authors:** Kristián Gulyás, Romana Gašparovičová, Monika Balogová, Natália Pipová, Petr Papežík, Jessica Hriňáková, Dalibor Uhrovič, Marcel Uhrin, Michal Benovics

**Keywords:** amphibia; helminth diversity; host-parasite dynamics; integrative taxonomy; nematoda; toads; erratum

Cambridge University Press regrets the omission of the the following in the above article

One of the ‘Supplementary Material’ files was mistakenly omitted on first publication, for which the Publishers apologise. The file for Supplementary Table 2 has now been uploaded.

The table legends were omitted from the article. The table legends are provided below.

Table 3: BoL = total body length; OeL = oesophagus length; NDA = neural ring distance from the anterior end; VDA = vulva distance from the anterior end; TaL = tail length; Ref = reference to the respective publication.

Table 4: BoL = total body length; BoW = body width; OeL = oesophagus length; BuL = bulbus length; BuW = bulbus width; NDA = neural ring distance from the anterior end; VuC = vulva characteristics; VPo = vulva position; VDA = vulva distance from the anterior end; EgS = egg shape; NPA = number of preanal papillae; SpC = spicule characteristics; SpL = spicule length; GuL = gubernaculum length; Ref = reference to the respective publication.

Table 5: BoL = total body length; BoW = body width; OeL = oesophagus length; BuL = bulbus length; BuW = bulbus width; NDA = neural ring distance from the anterior end; VuC = vulva characteristics; VPo = vulva position; VDA = vulva distance from the anterior end; EgS = egg shape; NPA = number of preanal papillae; SpC = spicule characteristics; SpL = spicule length; GuL = gubernaculum length; Ref = reference to the respective publication.

## References

[ref1] Gulyás K, Gašparovičová R, Balogová M, Pipová N, Papežík P, Hriňáková J, Uhrovič D, Uhrin M and Benovics M. A combined morphological and genetic survey of helminths in the European green toad *Bufotes viridis* (Laurenti, 1768) from eastern Slovakia. *Parasitology*. Published by Cambridge University Press, 6 October 2025. 10.1017/S0031182025100966. PMC12917419 41047570

