# Supplementary material for: A combined morphological and genetic survey of helminths in the European green toad Bufotes viridis (Laurenti, 1768) from eastern Slovakia
Source: Parasitology. 2025 Oct 6;152(13):1338–54. doi: 10.1017/S0031182025100966 (PMC12917419; doi:10.1017/S0031182025100966)
Supplement: Gulyás et al. supplementary material [file S0031182025100966sup001.docx]

| **Supplementary Table 1:** Recorded endohelminth species in green toads (*B. viridis* complex) over the last 70 years. | | | | | |
| --- | --- | --- | --- | --- | --- |
| Country | Source | Phylum | Family | Species | Localization in the host |
| Slovaka | Prokopič (1957) | Platyhelminthes Minot, 1876 | Telorchiidae Looss, 1899 | *Opisthioglyphe ranae* (Frölich, 1791) Looss, 1907 | intestines |
|  |  |  | Plagiorchiidae Lühe, 1901 | *Haplometra cylindracea* (Zeder, 1800) Looss, 1899 | lungs |
|  |  | Nematoda Diesing, 1861 | Molineidae Skryabin & Schulz, 1937 | *Oswaldocruzia bialata* (Molin, 1860) Travassos, 1917 | small intestines |
|  |  |  |  | *Oswaldocruzia subauricularis* (Rudolphi, 1819) | small intestines |
|  |  |  |  | *Oswaldocruzia ukrainae* Iwanitzky, 1928 | small intestines |
|  |  |  | Cosmocercidae Railliet, 1916 | *Cosmocerca ornata* (Dujardin, 1845) Diesing, 1861 | intestines |
|  |  |  | Rhabdiasidae Railliet, 1916 | *Rhabdias bufonis* (Schrank, 1788) Stiles & Hassall, 1905 | lungs |
|  | Kozák (1966) | Platyhelminthes Minot, 1876 | Pleurogenidae Looss, 1899 | *Pleurogenes claviger* (Rudolphi, 1819) Looss, 1899 | intestines |
|  |  |  | Polystomatidae Gamble, 1896 | *Polystoma integerrimum* (Frölich, 1791) Rudolphi, 1808 | urinary bladder |
|  | Kozák (1968) | Platyhelminthes | Polystomatidae | *Polystoma integerrimum* | urinary bladder |
|  |  |  | Plagiorchiidae | *Encyclometra colubrimurorum* (Rudolphi, 1819) Dollfus in Joyeux & Houdemer, 1928 | - |
|  |  | Nematoda | Rhabdiasidae | *Rhabdias bufonis* | lungs |
|  |  |  | Cosmocercidae | *Cosmocerca ornata* | intestines |
|  |  |  |  | *Cosmocerca commutata* (Diesing, 1851) Diesing, 1861 | intestines |
|  |  |  |  | *Aplectana acuminata* (Schrank, 1788) Railiet & Henry, 1916 | intestines |
|  |  |  |  | *Aplectana itzocanensis* Bravo Hollis, 1943 | intestines |
|  |  |  |  | **Aplectana linstowi* Yorke & Maplestone, 1926 (under the synonym *Aplectana kutassi* Ivanitzky, 1940) | intestines |
|  |  |  | Molineidae | *Oswaldocruzia bialata* | small intestines |
|  |  |  |  | *Oswaldocruzia goezi* Skrjabin & Schultz, 1952 | small intestines |
|  |  |  |  | *Oswaldocruzia iwanitzkyi* Sudarikov, 1951 | small intestines |
|  |  |  |  | *Oswaldocruzia subauricularis* | small intestines |
|  |  |  |  | *Oswaldocruzia ukrainae* | small intestines |
|  |  | Acanthocephala Rudolphi, 1808 | Paracanthocephalidae Golvan, 1960 | *Acanthocephalus ranae* (Schrank, 1788) Lühe, 1911 | intestines |
|  | Kozák (1969a) | Nematoda | Rhabdiasidae | *Rhabdias bufonis* | lungs |
|  |  |  | Cosmocercidae | *Cosmocerca ornata* | colon, cloaca |
|  |  |  |  | *Cosmocerca commutata* | cloaca |
|  |  |  |  | *Aplectana acuminata* | colon |
|  |  |  |  | *Aplectana itzocanensis* | colon |
|  |  |  |  | **Aplectana linstowi* (under the synonym *Aplectana kutassi*) | colon |
|  |  |  | Molineidae | *Oswaldocruzia bialata* | small intestines |
|  |  |  |  | *Oswaldocruzia goezi* | small intestines |
|  |  |  |  | *Oswaldocruzia iwanitzkyi* | small intestines |
|  |  |  |  | *Oswaldocruzia subauricularis* | intestines |
|  |  |  |  | *Oswaldocruzia ukrainae* | small intestines |
|  | Kozák (1969b) | Platyhelminthes | Pleurogenidae | *Pleurogenes claviger* | intestines |
|  |  |  | Polystomatidae | *Polystoma integerrimum* | urinary bladder |
|  |  |  | Haematoloechidae Freitas & Lent, 1939 | *Haematoloechus variegatus* (Rudolphi, 1819) Looss, 1899 | lungs |
|  |  |  | Plagiorchiidae | *Encyclometra colubrimurorum* | liver, peritoneum |
|  | Prokopič & Křivanec (1975) | Nematoda | Rhabdiasidae | *Rhabdias bufonis* | lungs |
|  |  |  | Cosmocercidae | *Oxysomatium brevicaudatum* (Zeder, 1800) Railliet & Henry, 1916 | intestines |
|  | Vojtková (1976) | Nematoda | Cosmocercidae | *Cosmocerca ornata* | big intestines |
|  |  |  |  | *Cosmocerca commutata* | big intestines |
|  |  |  | Rhabdiasidae | *Rhabdias bufonis* | lungs |
| Czechia | Vojtek and Vojtková (1975) | Platyhelminthes | Plagiorchiidae | *Encyclometra colubrimurorum* | liver, peritoneum |
|  |  |  |  | *Haplometra cylindracea* | - |
|  |  |  | Diplodiscidae Cohn, 1904 | *Diplodiscus subclavatus* (Goeze, 1782) Diesing, 1836 | - |
|  |  |  | Pleurogenidae | *Pleurogenes claviger* | - |
|  |  |  |  | *Pleurogenoides medians* (Olsson, 1876) Travassos, 1921 | - |
|  |  |  |  | *Prosotocus confusus* (Looss, 1894) Looss, 1899 | - |
|  |  |  | Haematoloechidae | *Haematoloechus variegatus* | - |
|  |  |  | Echinostomatidae Looss, 1899 | *Opisthioglyphe ranae (Frölich, 1791) Looss, 1907* | - |
|  | Vojtková (1976) | Nematoda | Cosmocercidae | *Cosmocerca ornata* | big intestines |
|  |  |  |  | *Cosmocerca commutata* | big intestines |
|  |  |  |  | *Aplectana acuminata* | big intestines, cloaca |
|  |  |  |  | *Aplectana itzocanensis* | big intestines, cloaca |
|  |  |  |  | **Aplectana linstowi* (under the synonym *Aplectana kutassi*) | big intestines, cloaca |
|  |  |  |  | *Oxysomatium brevicaudatum* | small intestines |
|  |  |  | Hedruridae Railliet, 1916 | *Hedruris androphora* Nitzsch, 1821 | stomach, lungs |
|  |  |  | Molineidae | *Oswaldocruzia filiformis* (Goeze, 1782) Skrjabin & Schultz, 1952 | small intestines |
|  |  |  |  | *Oswaldocruzia iwanitzkyi* | small intestines |
|  |  |  |  | *Oswaldocruzia ukrainae* | stomach, intestines |
|  |  |  | Rhabdiasidae | *Rhabdias bufonis* | lungs |
|  | Vojtková (1979) | Acanthocephala | Paracanthocephalidae | *Acanthocephalus falcatus* (Frölich, 1789) Lühe, 1911 | intestines |
|  |  |  |  | *Acanthocephalus ranae (Schrank, 1788) Lühe, 1911* | intestines |
|  | Vojtková (1980) | Platyhelminthes | Dilepididae Railliet & Henry, 1909 | *Nematotaenia dispar* (Goeze, 1782) Lühe, 1899 | intestines |
|  | Vojtková (1989) | Platyhelminthes | Polystomatidae | *Polystoma integerrimum* | urinary bladder |
| Belarus | Shimalov & Shimalov (2001) | Acanthocephala | Paracanthocephalidae | *Acanthocephalus ranae* | - |
|  |  | Nematoda | Acuariidae Railliet, Henry & Sisoff, 1912 | *Agamospirura* sp. | - |
|  |  |  | Cosmocercidae | *Aplectana acuminata* | - |
|  |  |  |  | *Cosmocerca ornata* | - |
|  |  |  | Molineidae | *Oswaldocruzia filiformis* | - |
|  |  |  | Rhabdiasidae | *Rhabdias bufonis* | - |
|  |  | Platyhelminthes | Gorgoderidae Looss, 1899 | *Gorgodera cygnoides* (Zeder, 1800) Looss, 1899 | - |
|  |  |  | Diplostomidae Poirier, 1886 | *Alaria alata* (Goeze, 1782) Krause, 1914 | - |
|  |  |  | Pleurogenidae | *Pleurogenes claviger* | - |
| Ukraine | Marushchak et al. (2024) | Nematoda | Cosmocercidae | *Cosmocerca commutata* | intestines, rectum |
|  |  |  | Molineidae | *Oswaldocruzia ukrainae* | intestines, rectum |
|  |  |  | Rhabdiasidae | *Rhabdias rubrovenosa* (Schneider, 1866) Semenov, 1929 | lungs |
|  |  | Platyhelminthes | Polystomatidae | *Polystoma viridis* Euzet, Combes & Batchvarov, 1974 | urinary bladder |
| Moldova | Gherasim & Erhan (2024) | Acanthocephala | Paracanthocephalidae | *Acanthocephalus ranae* | - |
|  |  |  | Pseudoacanthocephalidae Petrochenko, 1956 | *Pseudoacanthocephalus bufonis* (Shipley, 1903) Petrochenko, 1956 | - |
|  |  |  | Centrorhynchidae Van Cleave, 1916 | **Sphaerirostris picae* (Rudolphi, 1819) (under the synonym *Sphaerirostris teres* (Westrumb, 1821)) | - |
|  |  | Nematoda | Cosmocercidae | *Cosmocerca ornata* | - |
|  |  |  | Molineidae | *Oswaldocruzia filiformis* | - |
|  |  |  |  | *Oswaldocruzia duboisi* Ben-Slimane, Durette-Desset & Chabaud, 1993 | - |
|  |  |  | Spirocercidae Chitwood & Wehr, 1932 | *Spirocerca lupi* (Rudolphi, 1809) Chitwood, 1933 | - |
|  |  |  |  | *Ascarops strongylina* (Rudolphi, 1819) Alicata & McIntosh, 1933 | - |
|  |  |  | Acuariidae | *Agamospirura* sp. | - |
|  |  |  | Rhabdiasidae | *Rhabdias bufonis* | - |
|  |  | Platyhelminthes | Echinostomatidae | *Opisthioglyphe ranae* | - |
|  |  |  | Haematoloechidae | *Haematoloechus variegatus* | - |
|  |  |  | Gorgoderidae | *Gorgoderina vitelliloba* (Olsson, 1876) Looss, 1902 | - |
|  |  |  | Pleurogenidae | *Pleurogenes claviger* | - |
|  |  |  |  | *Pleurogenoides medians* | - |
|  |  |  |  | *Prosotocus confusus* | - |
|  |  |  | Diplodiscidae | *Diplodiscus subclavatus* | - |
|  |  |  | Strigeidae Railliet, 1919 | *Strigea sphaerula (*Rudolphi, 1803) Mathias, 1925 | - |
|  |  |  | Plagiorchiidae | *Haplometra cylindracea* | - |
|  |  |  | Diplostomidae | *Holostephanus volgensis* (Sudarikov, 1962) Vojtkova, 1966 | - |
|  |  |  |  | *Tylodelphys excavata* (Rudolphi, 1803) Szidat, 1935 | - |
|  |  |  | Polystomatidae | *Polystoma integerrimum* | - |
| European Russia | Kirillova et al. (2023) | Nematoda | Molineidae | *Oswaldocruzia ukrainae* | small intestines |
|  |  |  |  | *Oswaldocruzia filiformis* | small intestines |
| Türkiye | Yildirimhan (1999) | Acanthocephala | Paracanthocephalidae | *Acanthocephalus ranae* | small intestine |
|  |  | Nematoda | Cosmocercidae | *Cosmocerca commutata* | intestines |
|  |  |  |  | *Cosmocercoides* sp. | intestines |
|  |  |  | Rhabdiasidae | *Rhabdias bufonis* | lungs |
|  |  |  | Molineidae | *Oswaldocruzia filiformis* | small intestine |
|  |  | Platyhelminthes | Proteocephalidae La Rue, 1911 | *Proteocephalus* sp. | intestines |
|  |  |  | Nematotaeniidae Looss, 1910 | *Nematotaenia dispar* | intestines |
|  |  |  | Polystomatidae | *Polystoma viridis* | urinary bladder |
|  | Düşen and Oğuz (2010) | Nematoda | Cosmocercidae | *Cosmocerca ornata* | - |
|  |  |  |  | *Oxysomatium brevicaudatum* | - |
|  |  |  | Molineidae | *Oswaldocruzia filiformis* | - |
|  |  | Platyhelminthes | Pleurogenidae | *Pleurogenoides medians* | - |
|  |  |  | Nematotaeniidae | *Nematotaenia dispar* | - |
|  | Düşen et al. (2010) | Nematoda | Cosmocercidae | *Cosmocerca ornata* | - |
|  |  |  |  | *Oxysomatium brevicaudatum* | - |
|  |  |  | Molineidae | *Oswaldocruzia filiformis* | - |
|  |  | Platyhelminthes | Nematotaeniidae | *Nematotaenia dispar* | - |
|  |  |  | Polystomatidae | *Polystoma viridis* | - |
|  |  |  | Pleurogenidae | *Pleurogenoides medians* | - |
| Jordan | Al-Sorakhy & Amr (2003) | Platyhelminthes | Polystomatidae | *Polystoma viridis* | urinary bladder |
|  |  |  |  | *Polystoma integerrimum* | urinary bladder |
|  |  |  | Nematotaeniidae | *Nematotaenia chantalae* Dollfus, 1957 | - |
|  |  |  |  | *Nematotaenia dispar* | - |
| Iraq | Mohammad et al. (2010) | Nematoda | Cosmocercidae | *Cosmocerca commutata* | intestine |
|  |  |  |  | *Cosmocercoides variabilis* Harwood, 1930 | intestine |
|  |  |  | Rhabdiasidae | *Rhabdias bufonis* | lungs |
|  |  |  | Molineidae | *Oswaldocruzia filiformis* | intestine |
|  |  | Platyhelminthes | Proteocephalidae La Rue, 1911 | *Proteocephalus* sp. | small intestine |
|  |  |  | Nematotaeniidae | *Nematotaenia dispar* | small intestine |
| Iran | Rakhshandehroo et al. (2017) | Nematoda | Rhabdiasidae | *^†^Rhabdias ranae* Walton, 1929 | intestine |
|  |  | Platyhelminthes | Polystomatidae | *Polystoma viridis* | urinary bladder |
|  |  |  | Nematotaeniidae | *Nematotaenia dispar* | intestine |
| Uzbekistan | Vashetko & Siddikov (1999) | Acanthocephala | Paracanthocephalidae | *Acanthocephalus falcatus* | intestine |
|  |  |  |  | *Acanthocephalus ranae* | small intestine, stomach |
|  |  |  | Pseudoacanthocephalidae | *Pseudoacanthocephalus bufonis* | intestine |
|  |  |  |  | *Pseudoacanthocephalus bufonicola* (Kostylew, 1941) Petrotschenko, 1958 | intestine |
|  |  | Nematoda | Dioctophymidae Railliet, 1915 | *Hystrichis tricolor* Dujardin, 1845 | rectum |
|  |  |  | Cosmocercidae | *Cosmocerca commutata* | rectum |
|  |  |  |  | *Cosmocerca ornata* | rectum |
|  |  |  | Spirocercidae | *Ascarops strongylina* | stomach wall |
|  |  |  |  | *Physocephalus sexalatus* (Molin, 1860) | stomach wall |
|  |  |  |  | *Spirocerca lupi* | Inner organs |
|  |  |  | Gongylonematidae Sobolev, 1949 | *Gongylonema pulchrum* Molin, 1857 | stomach wall |
|  |  |  | Onchocercidae Leiper, 1911 | *Foleyella duboisi* (Gedoelst, 1916) | large intestine |
|  |  |  | Rhabdiasidae | *Rhabdias bufonis* | lungs |
|  |  |  | Molineidae | *Oswaldocruzia filiformis* | intestine |
|  |  |  |  | *Oswaldocruzia ukrainae* | intestines |
|  |  |  |  | *Oswaldocruzia bialata* | intestines |
|  |  | Platyhelminthes | Polystomatidae | *Polystoma integerrimum* | urinary bladder |
|  |  |  | Diplodiscidae | *Diplodiscus subclavatus* | intestine |
|  |  | Nematoda | Nematotaeniidae | *Nematotaenia dispar* | small intestine |
| * = Kozák (1969a) and Vojtková (1976) reports *A. linstowi* under the synonym *Aplectana kutassi* Ivanitzky, 1940, Gherasim & Erhan (2024) reports *Sphaerirostris picae* (Rudolphi, 1819) under the synonym *Sphaerirostris teres* (Westrumb, 1821); †=high probability of misidentification according to the included pictures. | | | | | |
