# Supplementary material for: A combined morphological and genetic survey of helminths in the European green toad Bufotes viridis (Laurenti, 1768) from eastern Slovakia
Source: Parasitology. 2025 Oct 6;152(13):1338–54. doi: 10.1017/S0031182025100966 (PMC12917419; doi:10.1017/S0031182025100966)
Supplement: Gulyás et al. supplementary material [file S0031182025100966sup002.docx]

| **Supplementary Table 2:** All the examined toads with the examined localities and the exact number of isolated nematodes divided into the corresponding families. | | | | | | | | |
| --- | --- | --- | --- | --- | --- | --- | --- | --- |
| ID | Loc_ID | Locality | Loc_Type | Coordinates | | Rhabdiasidae | Molineidae | Cosmocercidae |
| 110/24 | CE | Cestice | rural | 48.590383 | 21.100551 | 3 |  |  |
| 111/24 | CE | Cestice | rural | 48.590383 | 21.100551 | 3 |  | 2 |
| 112/24 | CE | Cestice | rural | 48.590383 | 21.100551 | 31 |  | 2 |
| 113/24 | CE | Cestice | rural | 48.590383 | 21.100551 | 22 |  | 12 |
| 114/24 | CE | Cestice | rural | 48.590383 | 21.100551 | 11 |  | 8 |
| 90/24 | CC | Čečejovce | rural | 48.598886 | 21.062267 | 7 |  | 25 |
| 91/24 | CC | Čečejovce | rural | 48.598886 | 21.062267 | 1 |  | 5 |
| 92/24 | CC | Čečejovce | rural | 48.598886 | 21.062267 | 8 |  | 3 |
| 93/24 | CC | Čečejovce | rural | 48.598886 | 21.062267 | 66 |  | 15 |
| 94/24 | CC | Čečejovce | rural | 48.598886 | 21.062267 |  | 1 | 19 |
| 147/24 | DE | Demjata | rural | 49.107776 | 21.311242 | 3 |  | 118 |
| 146/24 | CH | Chmeľovec | rural | 49.082935 | 21.371885 | 2 |  | 2 |
| 100/24 | KVP | Košice – KVP | urban | 48.715139 | 21.211511 |  |  | 205 |
| 101/24 | KVP | Košice – KVP | urban | 48.715139 | 21.211511 | 15 |  | 71 |
| 102/24 | KVP | Košice – KVP | urban | 48.715139 | 21.211511 |  |  | 103 |
| 103/24 | KVP | Košice – KVP | urban | 48.715139 | 21.211511 | 10 |  | 39 |
| 104/24 | KVP | Košice – KVP | urban | 48.715139 | 21.211511 | 33 | 1 |  |
| 01/21 | KVP | Košice – KVP | urban | 48.715139 | 21.211511 |  | 1 |  |
| 02/21 | KVP | Košice – KVP | urban | 48.715139 | 21.211511 |  | 4 |  |
| 03/21 | KVP | Košice – KVP | urban | 48.715139 | 21.211511 |  | 3 |  |
| 04/21 | KVP | Košice – KVP | urban | 48.715139 | 21.211511 |  |  |  |
| 05/21 | KVP | Košice – KVP | urban | 48.715139 | 21.211511 |  |  | 3 |
| 85/24 | MP | Košice – City Park | urban | 48.723888 | 21.265415 | 5 | 1 | 10 |
| 86/24 | MP | Košice – City Park | urban | 48.723888 | 21.265415 | 38 | 4 | 47 |
| 87/24 | MP | Košice – City Park | urban | 48.723888 | 21.265415 | 16 |  | 43 |
| 88/24 | MP | Košice – City Park | urban | 48.723888 | 21.265415 | 6 |  | 13 |
| 89/24 | MP | Košice – City Park | urban | 48.723888 | 21.265415 | 8 | 1 | 23 |
| 24/23 | MP | Košice – City Park | urban | 48.723888 | 21.265415 | 68 | 30 | 27 |
| 105/24 | ZP | Košice – City Park | urban | 48.718835 | 21.238002 | 6 |  | 28 |
| 106/24 | ZP | Košice – Zuzka's park | urban | 48.718835 | 21.238002 | 7 |  | 20 |
| 107/24 | ZP | Košice – Zuzka's park | urban | 48.718835 | 21.238002 | 10 |  | 9 |
| 108/24 | ZP | Košice – Zuzka's park | urban | 48.718835 | 21.238002 | 14 |  | 86 |
| 109/24 | ZP | Košice – Zuzka's park | urban | 48.718835 | 21.238002 | 15 |  | 51 |
| 95/24 | MK | Mokrance | rural | 48.593335 | 21.022273 | 11 |  | 35 |
| 96/24 | MK | Mokrance | rural | 48.593335 | 21.022273 | 5 |  | 14 |
| 97/24 | MK | Mokrance | rural | 48.593335 | 21.022273 | 24 |  |  |
| 98/24 | MK | Mokrance | rural | 48.593335 | 21.022273 | 22 |  | 3 |
| 99/24 | MK | Mokrance | rural | 48.593335 | 21.022273 | 5 |  |  |
| 142/24 | PH | Podhorany | rural | 49.083212 | 21.356049 | 2 |  | 18 |
| 143/24 | PH | Podhorany | rural | 49.083212 | 21.356049 |  |  | 6 |
| 144/24 | PH | Podhorany | rural | 49.083212 | 21.356049 | 6 |  | 3 |
| 145/24 | PH | Podhorany | rural | 49.083212 | 21.356049 |  | 4 | 100 |
| 137/24 | PKP | Prešov – City square | urban | 49.006469 | 21.224027 | 2 |  |  |
| 138/24 | PKP | Prešov – City square | urban | 49.006469 | 21.224027 | 5 |  |  |
| 139/24 | PKP | Prešov – City square | urban | 49.006469 | 21.224027 |  |  |  |
| 140/24 | PKP | Prešov – City square | urban | 49.006469 | 21.224027 |  |  |  |
| 141/24 | PKP | Prešov – City square | urban | 49.006469 | 21.224027 |  |  |  |
| 132/24 | PS | Prešov – Šváby | urban | 48.971488 | 21.266167 |  |  | 3 |
| 133/24 | PS | Prešov – Šváby | urban | 48.971488 | 21.266167 |  |  |  |
| 134/24 | PS | Prešov – Šváby | urban | 48.971488 | 21.266167 | 4 |  | 13 |
| 135/24 | PS | Prešov – Šváby | urban | 48.971488 | 21.266167 | 6 |  | 8 |
| 136/24 | PS | Prešov – Šváby | urban | 48.971488 | 21.266167 |  | 1 |  |
| 80/24 | RH | Rad site Hrušov | rural | 48.435857 | 21.861214 | 1 |  |  |
| 81/24 | RH | Rad site Hrušov | rural | 48.435857 | 21.861214 |  |  |  |
| 82/24 | RH | Rad site Hrušov | rural | 48.435857 | 21.861214 |  | 2 | 1 |
| 83/24 | RH | Rad site Hrušov | rural | 48.435857 | 21.861214 |  |  |  |
| 84/24 | RH | Rad site Hrušov | rural | 48.435857 | 21.861214 |  |  |  |
| 148/24 | VS | Vyšný Slivník | rural | 49.112080 | 21.275219 |  |  | 39 |
| 149/24 | VS | Vyšný Slivník | rural | 49.112080 | 21.275219 |  |  | 19 |
| 150/24 | VS | Vyšný Slivník | rural | 49.112080 | 21.275219 | 11 |  |  |
| 151/24 | VS | Vyšný Slivník | rural | 49.112080 | 21.275219 |  |  | 18 |

ID = the identification number of the toad, Locality = the name of the examined locality, Loc_ID = the abbreviation of the examined locality, Loc_Type = the defined type of locality (rural/urban).
